# Supplementary material for: Comprehensive Analysis of Rhodomyrtus tomentosa Chloroplast Genome
Source: Plants (Basel). 2019 Apr 4;8(4):89. doi: 10.3390/plants8040089 (PMC6524380; doi:10.3390/plants8040089)
Supplement: Supplementary file 1 [file plants-08-00089-s001.pdf]

Table 1. Codon usage of the *Rhodomyrtus tomentosa* chloroplast genome.

Amino acid	Codon	Count	RSCU	Amino acid	Codon	Count	RSCU
Phe	UUU(F)	966	1.29	Tyr	UAU(Y)	725	1.57
Phe	UUC(F)	529	0.71	Tyr	UAC(Y)	201	0.43
Leu	UUA(L)	859	1.89	Stop	UAA(*)	72	1.30
Leu	UUG(L)	549	1.21	Stop	UAG(*)	56	1.01
Leu	CUU(L)	568	1.25	His	CAU(H)	459	1.53
Leu	CUC(L)	200	0.44	His	CAC(H)	141	0.47
Leu	CUA(L)	370	0.81	Gln	CAA(Q)	689	1.54
Leu	CUG(L)	178	0.39	Gln	CAG(Q)	205	0.46
Ile	AUU(I)	1,046	1.44	Asn	AAU(N)	927	1.52
Ile	AUC(I)	445	0.61	Asn	AAC(N)	294	0.48
Ile	AUA(I)	687	0.95	Lys	AAA(K)	995	1.46
Met	AUG(M)	613	1.00	Lys	AAG(K)	369	0.54
Val	GUU(V)	470	1.38	Asp	GAU(D)	821	1.60
Val	GUC(V)	174	0.51	Asp	GAC(D)	204	0.40
Val	GUA(V)	512	1.51	Glu	GAA(E)	999	1.47
Val	GUG(V)	203	0.60	Glu	GAG(E)	363	0.53
Ser	UCU(S)	556	1.71	Cys	UGU(C)	202	1.41
Ser	UCC(S)	308	0.95	Cys	UGC(C)	84	0.59
Ser	UCA(S)	377	1.16	Stop	UGA(*)	38	0.69
Ser	UCG(S)	204	0.63	Trp	UGG(W)	460	1.00
Pro	CCU(P)	396	1.52	Arg	CGU(R)	327	1.29
Pro	CCC(P)	201	0.77	Arg	CGC(R)	83	0.33
Pro	CCA(P)	311	1.20	Arg	CGA(R)	351	1.39
Pro	CCG(P)	132	0.51	Arg	CGG(R)	105	0.41
Thr	ACU(T)	510	1.60	Ser	AGU(S)	389	1.20
Thr	ACC(T)	231	0.73	Ser	AGC(S)	113	0.35
Thr	ACA(T)	394	1.24	Arg	AGA(R)	456	1.80
Thr	ACG(T)	138	0.43	Arg	AGG(R)	198	0.78
Ala	GCU(A)	571	1.79	Gly	GGU(G)	548	1.34
Ala	GCC(A)	210	0.66	Gly	GGC(G)	157	0.38
Ala	GCA(A)	359	1.12	Gly	GGA(G)	655	1.60
Ala	GCG(A)	137	0.43	Gly	GGG(G)	281	0.68

RSCU: Relative Synonymous Codon Usage.

Table S2. Predicted RNA editing sites in the chloroplast genomes of *Rhodomyrtus tomentosa* by the PREP program

Gene	Nucleotide position	Codon change	Amino acid change	Score
accD	715	CTT-TTT	L-F	1.00
	794	TCG-TTG	S-L	0.80
	1,403	CCT-CTT	P-L	1.00
atpI	23	ACC-ATC	T-I	1.00
ccsA	89	TCA-TTA	S-L	1.00
matK	601	CAT-TAT	H-Y	1.00
	1,138	CGG-TGG	R-W	1.00
	1,148	TCA-TTA	S-L	0.86
	1,207	CAC-TAC	H-Y	1.00
ndhA	83	CCT-CTT	P-L	0.80
	313	CCT-TCT	P-S	1.00
	515	ACT-ATT	T-I	1.00
	554	ACA-ATA	T-I	1.00
	634	CCT-TCT	P-S	1.00
	746	CCT-CTT	P-L	1.00
	809	CCA-CTA	P-L	1.00
	995	TCA-TTA	S-L	1.00
	1,061	CCA-CTA	P-L	1.00
	1,082	TCA-TTA	S-L	1.00
ndhB	784	CTC-TTC	L-F	1.00
	905	TCA-TTA	S-L	1.00
	1,223	CCA-CTA	P-L	1.00
	1,342	CAT-TAT	H-Y	1.00
	1,367	TCA-TTA	S-L	0.80
	1,502	TCT-TTT	S-F	1.00
ndhD	2	ACG-ATG	T-M	1.00
	313	CGG-TGG	R-W	0.80
	383	TCA-TTA	S-L	1.00
	1,405	CTT-TTT	L-F	0.80
ndhF	160	CTT-TTT	L-F	1.00
	259	CAC-TAC	H-Y	1.00
	586	CTT-TTT	L-F	0.80
	1,420	CAT-TAT	H-Y	1.00
	2,231	TCC-TTC	S-F	1.00
ndhG	166	CAT-TAT	H-Y	0.80
	314	ACA-ATA	T-I	0.80
psaI	83	TCT-TTT	S-F	0.86
psbE	214	CCT-TCT	P-S	1.00
psbF	77	TCT-TTT	S-F	1.00
